# Adult separation anxiety in patients with complicated grief versus healthy control subjects: relationships with lifetime depressive and hypomanic symptoms

**DOI:** 10.1186/1744-859X-10-29

**Published:** 2011-10-27

**Authors:** Liliana Dell'Osso, Claudia Carmassi, Martina Corsi, Irene Pergentini, Chiara Socci, Angelo GI Maremmani, Giulio Perugi

**Affiliations:** 1 Department of Psychiatry, Neurobiology, Pharmacology and Biotechnology, University of Pisa, Pisa, Italy

## Abstract

**Background:**

Around 9% to 20% of bereaved individuals experience symptoms of complicated grief (CG) that are associated with significant distress and impairment. A major issue is whether CG represents a distinctive nosographic entity, independent from other mental disorders, particularly major depression (MD), and the role of symptoms of adult separation anxiety. The purpose of this study was to compare the clinical features of patients with CG versus a sample of healthy control subjects, with particular focus on adult separation anxiety and lifetime mood spectrum symptoms.

**Methods:**

A total of 53 patients with CG and 50 healthy control subjects were consecutively recruited and assessed by means of the Structured Clinical Interview for DSM-IV Axis-I disorders (SCID-I/P), Inventory of Complicated Grief (ICG), Adult Separation Anxiety Questionnaire (ASA-27), Work and Social Adjustment Scale (WSAS) and Mood Spectrum-Self Report (MOODS-SR) lifetime version.

**Results:**

Patients with CG reported significantly higher scores on the MOODS-SR, ASA-27, and WSAS with respect to healthy control subjects. The scores on the ASA-27 were significantly associated with the MOODS-SR depressive and manic components amongst both patients and healthy control subjects, with a stronger association in the latter.

**Conclusions:**

A major limitation of the present study is the small sample size that may reduce the generalizability of the results. Moreover, lifetime MOODS-SR does not provide information about the temporal sequence of the manic or depressive symptoms and the loss. The frequent comorbidity with MD and the association with both depressive and manic lifetime symptoms do not support the independence of CG from mood disorders. In our patients, CG is associated with high levels of separation anxiety in adulthood. However, the presence of lifetime mood instability, as measured by the frequent presence of depressive and hypomanic lifetime symptoms, suggests that cyclothymia might represent the common underlying feature characterizing the vulnerability to both adult separation anxiety and CG.

## Background

A growing body of literature provides evidence that a minority of individuals (9% to 20%) that experience the loss of a relative or a significant other may report symptoms of unresolved grief that are associated with significant distress and impairment, heightened risk for depression, anxiety, alcohol and tobacco consumption, and suicidal ideation [1-8]. Increasing amounts of research have been focused on identifying the specific set of psychiatric symptoms that characterize this condition, corroborating the need to include this syndrome in the forthcoming *Diagnostic and Statistical Manual of Mental Disorders*, fifth edition (DSM-V), as a distinctive diagnosis. Complicated grief (CG) is identified by symptoms of both separation and traumatic distress [4,5,9,10] which are distinctive from other Axis I mental disorders, primarily major depression (MD) [11-15].

Vulnerability to CG has been rooted in attachment disturbances [16-19]. Van Doorn *et al. *found that CG symptoms were significantly associated with an insecure attachment style among caregivers of terminally ill spouses [16]. Fraley and Bonanno subsequently reported that an anxious, as opposed to secure or dismissively avoidant, attachment style was associated with chronic grief after a loss [18]. Silverman and collaborators found that childhood abuse and serious neglect were significantly associated with CG in widowhood, suggesting that early childhood experiences that disrupt primary attachment bonds can determine long lasting vulnerability to CG [17]. In line with these studies, Vanderverker and collaborators first explored symptoms of separation anxiety in childhood as predictors of CG onset in adulthood [20]. Nevertheless, to the best of our knowledge, no study to date has explored symptoms of adult separation anxiety amongst patients with CG with respect to healthy subjects. Thus, we hypothesized that bereaved individuals who present with CG will be those who report elevated levels of separation anxiety in adulthood.

Separation anxiety (SA) is included in the fourth edition of the DSM (DSM-IV) among childhood and adolescent disorders, and it has been typically described in juvenile population as an antecedent of mood and anxiety disorders. An adult form of excessive and often disabling distress in the face of actual or perceived separation from major attachment figures has been recently described [21]; in particular juvenile and adult separation anxieties have been associated with bipolar spectrum disorders, specifically with mood instability of cyclothymic type [22-24].

The aim of the present study was to investigate the presence of adult separation anxiety and lifetime mood spectrum symptoms amongst patients with CG with respect to healthy control subjects.

## Methods

### Study sample

A consecutive sample of 53 outpatients was recruited as part of a multicenter Italian study aimed at assessing the validity and reliability of a new instrument for assessing the trauma and loss spectrum [25,26]. Eligible patients presented with a diagnosis of CG, as assessed by a total score higher than 25 on the Inventory of Complicated Grief (ICG) [27], referring to a loss that had occurred at least 6 months before entering the study. All patients were taking psychotropic medications. Exclusion criteria were neurological diseases, or the presence of psychotic symptoms at the time of the assessments. A sample of 50 healthy control subjects, age and gender matched with CG patients, was recruited from the general population. Healthy control subjects did not fulfill the criteria for current or lifetime Axis I psychiatric disorders. Further, they had experienced the loss of a close friend or a relative but their total score on the ICG referring to this loss was lower than 25. Thus, both CG patients and healthy control subjects had experienced the loss of a close friend or a relative.

The Ethics Committee of the University of Pisa approved all recruitment and assessment procedures. Eligible subjects provided written informed consent after receiving a complete description of the study and having an opportunity to ask questions.

The assessment tools utilized included the Structured Clinical Interview for DSM-IV Axis-I disorders (SCID-I/P) [28], the Adult Separation Anxiety Questionnaire (ASA-27) [29], the Work and Social Adjustment Scale (WSAS) [30], and the Mood Spectrum-Self Report (MOODS-SR) lifetime version [31].

### Assessment instruments

The SCID-I/P was administered by psychiatrists trained and certified in the use of all instruments in this study.

The ICG is a self-report instrument that can be used to identify CG when the total score is > 25, that demonstrated high internal consistency (Cronbach's α = 0.94), and convergent and criterion validity [27]. The ICG total score also showed a fairly high association with the Beck Depression Inventory (BDI) [32] total score (r = 0.67, *P *< 0.001), the Texas Revised Inventory of Grief (TRIG) [33] score (r = 0.87, *P *< 0.001), and the Grief Measurement Scale (GMS) [34] score (r = 0.70, *P *< 0.001).

The ASA-27 is a self-report measure developed to rate separation anxiety symptoms in adult life (from 18 years of age). Principal components analysis revealed a coherent construct of adult separation anxiety with high internal consistency (Cronbach's α = 0.95) and sound test-retest reliability (r = 0.86; *P *< 0.001). Further, a receiver operation characteristic (ROC) analysis against the semistructured interview yielded a high area under the curve (AUC) index of 0.9, suggesting that the questionnaire is an adequate alternative measure of adult separation anxiety [29].

The WSAS is a self-report scale of functional impairment that includes five questions rating interference of psychiatric symptoms in work, home management, social or private leisure activities, and ability to form and maintain close relationships with others. Each question is rated on a 0 to 8 scale with 0 indicating no impairment at all and 8 indicating very severe impairment. The Cronbach's α measure of internal scale consistency ranged from 0.70 to 0.94, the test-retest correlation was 0.73, and the WSAS interactive voice response administrations gave correlations of 0.81 and 0.86 with clinician interviews [30]. Correlations of WSAS with severity of depression and obsessive-compulsive disorder symptoms were 0.76 and 0.61, respectively, and the scores were sensitive to patient differences in disorder severity and treatment-related change [30].

The MOODS-SR is an instrument developed and validated to assess lifetime mood spectrum symptoms [35], including manic and depressive features, rhythmicity and vegetative functions. The manic and depressive components are subtyped into mood, energy and cognition domains, focusing on manic or depressive symptoms, respectively. The rhythmicity and vegetative functions domain include changes in energy, physical wellbeing, mental and physical efficiency, related to the weather and season, and changes in appetite, sleep and sexual activities. The sum of the scores on the 3 manic domains (mood, energy, cognition) constitutes the 'manic component' (62 items) and that of the 3 depressive domains the 'depressive component' (63 items). The rhythmicity and vegetative functions domain includes 29 items. The instrument can be downloaded from http://www.spectrum-project.org.

### Statistical analyses

Comparisons of familial, epidemiological, clinical and course characteristics between the two subgroups were conducted using unpaired Student's t-test for dimensional variables (or Mann-Whitney test when appropriate) and χ^2 ^test for categorical ones (or Fisher exact test when appropriate). Considering the number of comparisons and the number of subjects in each group, our results are prone to both type I and type II errors. However, given the exploratory nature of our study, we decided to set a two-tailed significance level at *p *< 0.01. Pearson's correlations between the total scores of the ASA-27, WSAS and MOODS-SR, and those of the manic and depressive components of the MOODS-SR were also calculated. All statistical analyses were carried out using SPSS (SPSS Inc., Chicago, IL, USA), V. 15.0 [36].

## Results

The study sample included a total of 103 subjects: 53 patients with CG (46 women and 7 men) and 50 healthy control subjects (44 women and 6 men). Demographic characteristics of the study samples are reported in Table 1.

**Table 1:** Demographic characteristics of the study participants

	Complicated grief (N = 53)	Healthy control subjects (N = 50)	t	*P *value
Age	50.17 ± 14.57	51.42 ± 13.60	0.91	0.78
	N (%)	N (%)	χ^2^	
Women	46 (87)	44 (88)	0.03	0.85
Men	7 (13)	6 (12)		

Marital status:				
Single	12 (22.6)	11 (22)	8.68	0.034
Married/living with partner	24 (45.3)	34 (68)		
Widows/widowers	15 (28.3)	5 (10)		
Separated/divorced	2 (3.8)	0		

Education level:				
Primary school	13 (24.5)	4 (8)	12.8	0.002
High school	33 (62.3)	25 (50)		
University degree	7 (13.2)	21 (42)		

Occupation:				
Employed full/part time	25 (47.1)	30 (60)	4.6	0.02
Unemployed	7 (13.2)	3 (6)		
Retired	17 (32.1)	10 (20)		
Other	4 (7.5)	7 (4)		

CG patients and healthy control subjects reported a mean age of 50.17 ± 14.57 years and 51.42 ± 13.60 years, respectively. Almost half of the patients with CG (N = 23, 46%) and more than half of healthy control subjects (N = 34, 68%) were married or living with their partner. As expected, the majority of patients with CG were widows or widowers (N = 13, 26%) in comparison with healthy control subjects (N = 5, 10%). Patients with CG reported the loss of a spouse/partner (N = 19, 35.8%), brother/sister (N = 6, 11.3%), child (N = 4, 7.6%), parent (N = 8, 15.1%), or close friend (N = 16, 30.2%).

A total of 38 CG patients (71.7%) fulfilled DSM-IV text revision (DSM-IV-TR) criteria for MD, 5 (9.4%) for bipolar disorder (BD) of type II and only 1 (1.8%) for BD of type I. Other current Axis-I diagnoses were: post-traumatic stress disorder (PTSD) (N = 27, 50.9%), panic disorder (PD) (N = 14, 26.4%), generalized anxiety disorder (GAD) (N = 6, 11.3%), social anxiety disorder (SAD) (N = 3, 5.6%), and alcohol abuse (N = 2, 3.7%). Multiple diagnoses were common: 13 (24.5%) patients met criteria for MD and PTSD, 11 (20.7%) for both MD and PD, and 4 (7.5%) for both BD and PD. Only one patient had four diagnoses, specifically BD, alcohol abuse, GAD and PD.

Patients with CG and healthy control subjects reported a mean ICG total score of 38.68 ± 10.15 and 10.06 ± 9.42. The clinical characteristics of the study samples, including mean ± SD scores on the ASA-27, MOODS-SR (total, depressive and manic components) and WSAS are reported in Table 2.

**Table 2:** Clinical characteristics of the study participants

	Complicated grief (N = 53), mean ± SD	Healthy control subjects (N = 50), mean ± SD	t	*P *value
ASA-27	26.77 ± 15.68	12.80 ± 8,98	-5.51	0.000
MOODS-SR:				
Total	65.34 ± 27.11	37.50 ± 22.02	-5.7	0.000
Depressive component	30.19 ± 14.04	11.24 ± 9.59	-7.95	0.000
Manic component	17.26 ± 11.14	11.24 ± 10.43	-2.83	0.006
WSAS	17.42 ± 10.12	3.51 ± 5.50	28.39	0.000

ASA-27 = Adult Separation Anxiety Questionnaire; MOODS-SR = Mood Spectrum-Self Report; WSAS = Work and Social Adjustment Scale.

Patients with CG reported significantly higher total scores (*p *= 0.000) in all the assessments with respect to healthy control subjects. Further, for the MOODS-SR components, patients reported significantly higher scores on the depressive (*p *= 0.000) and manic (*p *= 0.006) components with respect to healthy control subjects.

Within the sample of patients with CG, significant positive correlations were reported between the depressive component of the MOODS-SR and the total scores of the ASA-27 (*p *= 0.022) and WSAS (*p *= 0.024). Further, a positive significant (*p *= 0.037) correlation was found between the MOODS-SR manic component and the ASA-27 total score. Highly significant positive correlations (*p *= 0.000) between either the depressive or the manic components of the MOODS-SR and the ASA-27 total scores were also found within the group of healthy control subjects.

## Discussion

Before discussing the results of the present study, several limitations should be taken into account. The small sample size may represent a major limitation. Further, the lifetime MOODS-SR does not provide information about the severity and the temporal sequence of the manic or depressive symptoms, as well as their relationships with the loss. Further, we did not recruit a comparison sample of patients with major depression to compare with those with CG.

Consistently with previous reports, patients with CG reported high prevalence of mood disorders, mainly MD (about 70%) but also BD (about 10%). Other common comorbid conditions were PTSD, PD and GAD.

A major issue in defining CG as an independent diagnostic category has been its distinction from mood disorders, particularly MD [12,13,15]. Our results confirm that distinguishing CG and MD may be difficult because of the frequent (80%) co-occurrence of the two conditions [1,12]. Similar findings have also been reported in other studies in which rates of comorbidity between CG and MD ranged from 52 to 70% [13,37].

A significant proportion (almost 10%) of our patients reported bipolar comorbidity. Interestingly, high rates (24%) of CG comorbidity have been found in individuals with bipolar disorder [13,38], and its presence was associated with additional psychiatric comorbidity, greater bipolar disorder severity and functional impairment, and lifetime suicide attempts.

As expected, CG patients reported more severe adult separation anxiety symptoms in comparison with matched healthy control subjects. This finding is consistent with previous reports of separation anxiety in childhood as predictors of CG onset in adulthood [20] and with the idea that vulnerability to CG is related to attachment disturbances [16-19]. Thus, our results seem to corroborate the hypothesis that bereaved individuals who develop CG will be those who present elevated levels of separation anxiety in adulthood. Nevertheless, these data do not permit any inference on the independence of CG diagnosis from mood disorders, as its significant correlations with MOODS-SR score seem to indicate a relationship with both depressive and manic lifetime symptoms. In other words, lifetime mood instability (cyclothymia or cyclothymic temperament) might represent the common underlying feature characterizing the vulnerability to both conditions (adult separation anxiety and CG). Recent reports on mood disorder patients found a strong relationship between cyclothymic mood instability and separation anxiety [22,24]. Of note, in our sample lifetime mood symptoms of both polarities were strongly related to adult separation anxiety scores in healthy control subjects as well. These findings suggest that mood variations and separation anxiety are related in a dimensional way. It is possible that, in healthy control subjects, the positive correlation between separation anxiety and lifetime mood symptoms of both polarities is even stronger because it is not confounded by the presence of symptoms related to the presence of the affective disorder. The anxious-cyclothymic connection has been observed not only in Latino cultures but in many others as well [39].

As already mentioned, the lack of a comparative group of patients with MD is a major limitation of this study, thus a comparison with MD patients on adult separation anxiety and lifetime mood instability is warranted in order to further clarify the independence and the possible relationships of CG from other mood disorders.

## Conclusions

In summary, in our patients CG was associated with high levels of separation anxiety in adulthood and with frequent comorbid MD. However, the presence of lifetime mood instability, as measured by the frequent presence of depressive and hypomanic lifetime symptoms, suggests that cyclothymia might represent the common underlying feature characterizing the vulnerability to both adult separation anxiety and CG.

## Competing interests

The authors declare that they have no competing interests.

## Authors' contributions

LD, CC and GP conceived the study, participated in its design, recruitment and data analyses, and drafted the manuscript. MC, IP, CS, AIM took part in the recruitment phase and the data analyses. All authors revised the manuscript for intellectual content, read and approved the final manuscript
